# Space: reconciling multiple spatial domain identification algorithms via consensus clustering

**DOI:** 10.1093/bioadv/vbaf084

**Published:** 2025-04-11

**Authors:** Daoliang Zhang, Wenrui Li, Xinyi Sui, Na Yu, Shan Wang, Zhiping Liu, Xiaowo Wang, Zhiyuan Yuan, Rui Gao, Wei Zhang

**Affiliations:** Center of Intelligent Medicine, School of Control Science and Engineering, Shandong University, Jinan, Shandong 250061, China; MOE Key Lab of Bioinformatics and Bioinformatics Division of BNRIST, Department of Automation, Tsinghua University, Beijing 100084, China; Center of Intelligent Medicine, School of Control Science and Engineering, Shandong University, Jinan, Shandong 250061, China; Center of Intelligent Medicine, School of Control Science and Engineering, Shandong University, Jinan, Shandong 250061, China; Center of Intelligent Medicine, School of Control Science and Engineering, Shandong University, Jinan, Shandong 250061, China; Center of Intelligent Medicine, School of Control Science and Engineering, Shandong University, Jinan, Shandong 250061, China; MOE Key Lab of Bioinformatics and Bioinformatics Division of BNRIST, Department of Automation, Tsinghua University, Beijing 100084, China; Institute of Science and Technology for Brain-Inspired Intelligence, Center for Medical Research and Innovation, Shanghai Pudong Hospital, Fudan University Pudong Medical Center, Fudan University, Shanghai 200433, China; Center of Intelligent Medicine, School of Control Science and Engineering, Shandong University, Jinan, Shandong 250061, China; Center of Intelligent Medicine, School of Control Science and Engineering, Shandong University, Jinan, Shandong 250061, China

## Abstract

**Motivation:**

The rapid development of spatially resolved transcriptomics (SRT) technologies has provided unprecedented opportunities for characterizing and understanding tissue architecture. As this field continues to advance, various methods have been developed to computationally identify spatial domains within tissues. However, the performance of different algorithms on the same dataset is not always consistent. This inconsistency makes it difficult for researchers to select the most reliable results for downstream analysis.

**Results:**

To address this challenge, we propose a domain identification method named Space. Space measures consistency between different methods to select reliable algorithms. It then constructs a consensus matrix to integrate the outputs from multiple algorithms. We introduce similarity loss, spatial loss, and low-rank loss in Space to enhance the accuracy and optimize computational efficiency. This strategy not only resolves the inconsistent issue of clustering labels among different methods but also achieves highly reliable clustering output. Flexible interfaces are also provided for downstream analysis such as visualization, domain-specific gene analysis and trajectory inference. Testing results on multiple publicly available SRT datasets demonstrate that Space performs exceptionally well in deciphering key tissue structures and biological features.

**Availability and implementation:**

The Space package can be easily installed through conda or mamba, and its source code is available at https://honchkrow.github.io/Space.

## 1 Introduction

Characterizing tissue regions from a spatial perspective is essential for deciphering tissue development and understanding biological mechanisms. The continuous advancement of spatially resolved transcriptomics (SRT) technologies has provided a robust data foundation, enabling the identification of structural patterns within tissue. In recent years, various computational methods have been proposed for identifying spatial domains in SRT data. For instance, STAGATE (Dong and Zhang 2022) and MENDER (Yuan 2024) integrate gene expression information with spatial locations to decipher tissue structure. Methods like stLearn (Pham *et al.* 2023) and DeepST (Xu *et al.* 2022) perform multi-modal clustering analysis by combining gene expression profiles, spatial locations, and morphological features.

There is no doubt that each of these methods has its own unique strategies and characteristics. However, the performance of different algorithms on the same dataset is not always consistent. To address this problem, we propose a spatial domain identification framework called Space. In Space, we integrate 10 widely-used spatial domain identification methods. Additionally, we employ consensus clustering to combine the outputs of the reliable methods, achieving optimal domain identification results. To address the challenge of determining the number of spatial domains in unlabeled datasets, we propose a strategy for estimating the number of spatial domains. Space also offers a variety of evaluation metrics and user-friendly interfaces for downstream analysis.

## 2 Materials and methods

**Figure 1.**
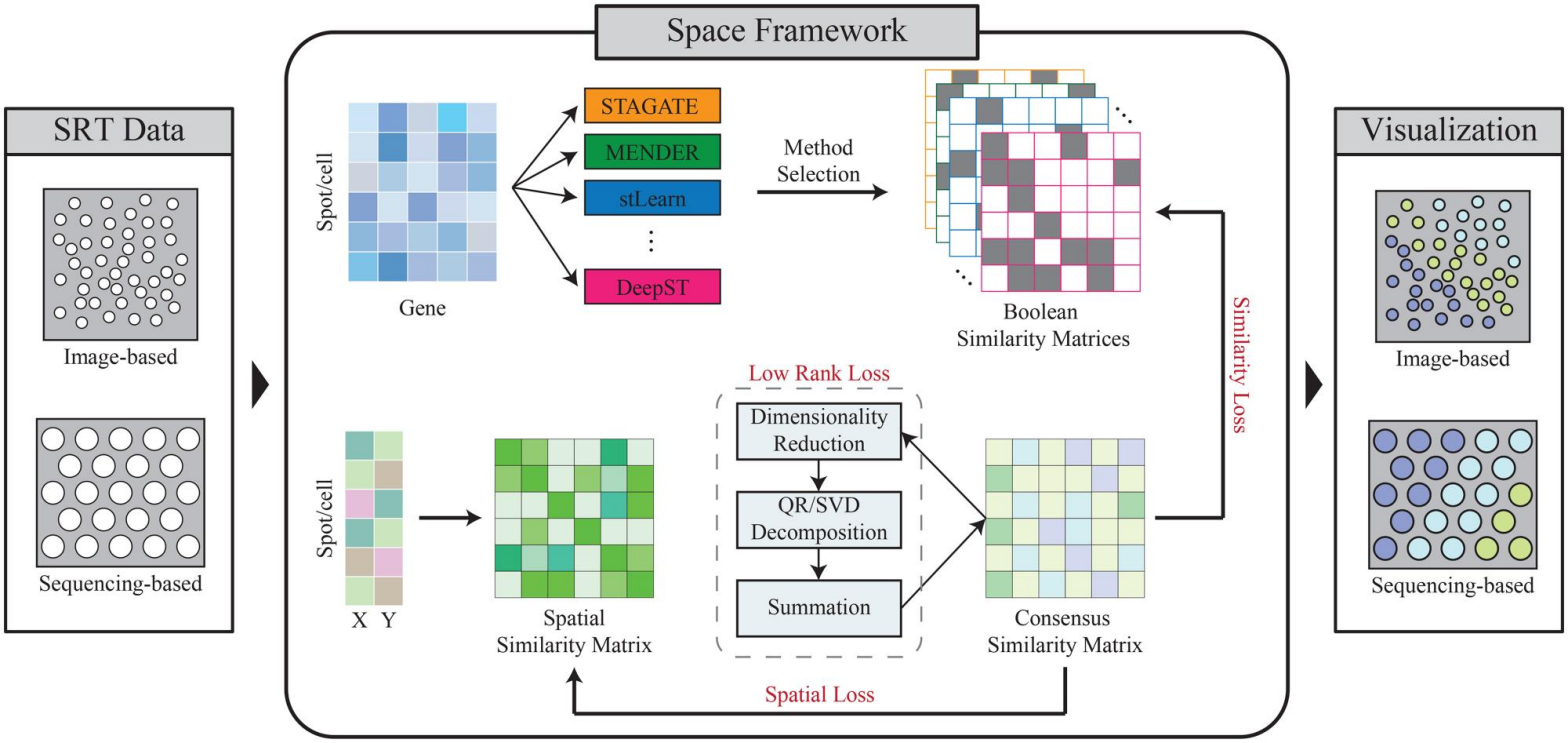
The workflow of Space.

The flowchart of Space is shown in Fig. 1. First, we select ten methods based on their performance, popularity, and methodological diversity, and applied them to the SRT datasets. Next, we identify algorithms with consistent results and convert these results into Boolean similarity matrices which represent whether different cells are classified into the same spatial domain. Simultaneously, considering that spatially adjacent cells typically share functional similarities, we introduce a spatial similarity matrix. Next, Space constructs a consensus similarity matrix and minimizes the similarity loss and spatial loss with the two aforementioned matrices respectively to obtain the consensus result. To reduce noise and improve model stability, Space also incorporates a low-rank loss through nuclear norm constraint. Finally, spectral clustering is adopted to identify spatial domains. For more detailed information, please see Supplementary Materials.

We conducted comprehensive tests on SRT datasets from different platforms. The experimental results demonstrate that Space effectively combines the strengths of different algorithms, obtaining precise and robust results (Supplementary Figs S1–S4).

For user convenience, Space offers a simple deployment process. The entire functionality can be executed with a few Python commands. Additionally, Space supports input and output compatibility with current mainstream analysis software, such as SCANPY (Wolf *et al.* 2018), enabling seamless downstream analyses for users from different fields.

## 3 Conclusion

Here, we propose Space as a highly integrated spatial domain identification toolkit. Users can run it without the need for complex parameter tuning. This toolkit not only helps researchers quickly decipher tissue structures but also facilitates downstream analysis of SRT data in future studies.

## Supplementary Material

vbaf084_Supplementary_Data

## Data Availability

The datasets used this article are public available and listed in the supplementary material.
