# Supplementary material for: Space: reconciling multiple spatial domain identification algorithms via consensus clustering
Source: Bioinform Adv. 2025 Apr 11;5(1):vbaf084. doi: 10.1093/bioadv/vbaf084 (PMC12037102; doi:10.1093/bioadv/vbaf084)
Supplement: vbaf084_Supplementary_Data [file vbaf084_supplementary_data.docx]

Supplementary Materials

# The Model of the Space

Space employs consensus clustering to integrate multiple methods, enhancing the accuracy and efficiency of spatial domain identification from SRT data.

**Obtaining Results from Different Methods**

Suppose we have an SRT data containing $m$ spots (or cells). We first use $n$ different spatial domain identification methods to obtain clustering results $C_{i}$, where $i=1,2,3...,n$. Space provides 10 state-of-the-art (SOTA) methods: GraphST (Long *et al.*, 2023), Leiden (Traag *et al.*, 2019), MENDER (Yuan, 2024), Louvain (Blondel *et al.*, 2008), SEDR (Xu *et al.*, 2024), SpaceFlow (Ren *et al.*, 2022), SpaGCN (Hu *et al.*, 2021), STAGATE (Dong and Zhang, 2022), stGCL (Yu *et al.*, 2023), and stLearn (Pham *et al.*, 2020). Users can choose and integrate any combination of these methods.

**Filtering Low-Consistency Methods**

Next, we assess the consistency of each method by calculating the Adjusted Rand Index (ARI) between different clustering results. Given two clustering results, $U$ and $V$, we construct a confusion matrix $\boldsymbol{C}$, where $C_{ij}$ represents the number of sample pairs that belong to the $i_{th}$ cluster in $U$ and the $j_{th}$ cluster in $V$. Then, based on the confusion matrix $\boldsymbol{C}$, we can calculate the following metrics.

$$a=\sum_{i,j} \left( \begin{matrix} C_{ij} \\ 2 \end{matrix} \right)$$

$$b=\sum_{i} \left( \begin{matrix} \sum_{j} C_{ij} \\ 2 \end{matrix} \right)$$

$$c=\sum_{j} \left( \begin{matrix} \sum_{i} C_{ij} \\ 2 \end{matrix} \right)$$

$$d=\left( \begin{matrix} n \\ 2 \end{matrix} \right)$$

Here, $a$ represents the number of sample pairs that are completely consistent between the clustering results. $b$ and $c$ represent the number of sample pairs that are consistent in $U$ and $V$, respectively. $d$ is the total number of possible sample pairs. Then, we can calculate ARI:

$$ARI=\frac{a-\frac{b\cdot c}{d}}{\frac{b+c}{2}-\frac{b\cdot c}{d}}$$

Based on the ARI value, we can generate an $n\times n$ similarity matrix $\boldsymbol{T}$, where $T_{ij}$ denotes the ARI value for method $i$ and $j$. By calculating the mean of each column in the similarity matrix $\boldsymbol{T}$, we can determine the average ARI for each method. We then apply a threshold $\alpha$ to filter the average ARI, retaining only the $K$ methods with higher consistency for subsequent result integration.

$$mean\_ARI_{i}=\frac{1}{n}\sum_{j=1}^{n} T_{ij}$$

$$retain\_methods=\left\{ i|mean\_ARI_{i}\geq\alpha\right\}$$

**Constructing Similarity Matrix for Each Method**

Next, we construct a similarity matrix $\boldsymbol{B}_{\boldsymbol{i}}$ for each of the retained $K$ methods, where $i=1,\ldots,K$. This matrix represents the consistency of the clustering results of different spots. Each element $\boldsymbol{B}_{\boldsymbol{i}}(p,q)$ in $\boldsymbol{B}_{\boldsymbol{i}}$ indicates the similarity between spot $p$ and spot $q$ using method $i$. If the two spots are clustered into the same category by method $i$, then $\boldsymbol{B}_{\boldsymbol{i}}(p,q)$ is assigned a value of 1. Otherwise, $\boldsymbol{B}_{\boldsymbol{i}}(p,q)$ is assigned a value of 0.

$$\boldsymbol{B}_{\boldsymbol{i}}=\left[ \begin{matrix} b_{(1,1)} & \cdots& b_{(1,m)} \\ \vdots& \ddots& \vdots\\ b_{(m,1)} & \cdots& b_{(m,m)} \end{matrix} \right]$$

where $m$ is the number of spot.

**The Construction of the Spatial Similarity Matrix**

Cells that are closer in spatial distance often exhibit functional similarity. Therefore, to account for spatial location, we construct a spatial similarity matrix $\boldsymbol{S}$ by calculating the Euclidean distance. Given that the spatial coordinates of spots $s_{i}$ and $s_{j}$​ are $(l_{xi},l_{yi}) and (l_{xj}, l_{yj})$, respectively, their Euclidean distance is calculated as follows:

$$d\left( s_{i},s_{j} \right)=\sqrt{\left( l_{xi}-l_{xj} \right)^{2}+\left( l_{yi}-l_{yj} \right)^{2}}.$$

Furthermore, we calculate the spatial similarity matrix $\boldsymbol{S}$ using a Gaussian kernel.

$$\boldsymbol{S}_{ij}=\exp\left( -\frac{d\left( s_{i},s_{j} \right)^{2}}{2\sigma^{2}} \right)$$

Here, $\sigma$ is the bandwidth of the Gaussian kernel. The matrix $\boldsymbol{S}$ will be used for the calculation of spatial loss.

**Loss Function and Model Optimization**

We randomly generate an $m\times m$ consensus similarity matrix $\boldsymbol{H}$. Then, we use the Mean Squared Error (MSE) to measure the difference between $\boldsymbol{B}$, $\boldsymbol{S}$ and $\boldsymbol{H}$.

For method $i$, the similarity loss is defined as:

$$MSE(\boldsymbol{B}_{\boldsymbol{i}},\boldsymbol{H})=\frac{1}{m^{2}}\sum_{p=1}^{m} \sum_{q=1}^{m} {(\boldsymbol{B}_{\boldsymbol{i}}(p,q)-\boldsymbol{H}(p,q))}^{2}.$$

The overall similarity loss is

$$L_{methods}=\frac{1}{n}(\sum MSE(\boldsymbol{B}_{\boldsymbol{i}}, \boldsymbol{H})).$$

The spatial loss is defined as

$$L_{Spatial}=MSE(\boldsymbol{S},\boldsymbol{H})=\frac{1}{m^{2}}\sum_{p=1}^{m} \sum_{q=1}^{m} {(\boldsymbol{S}(p,q)-\boldsymbol{H}(p,q))}^{2}.$$

To reduce noise and decrease model complexity, we generate a random matrix $O$ to map the consensus similarity matrix $H$ to a lower-dimensional space. The mapped matrix $Y$ is represented as follows:

$$\boldsymbol{Y}=\boldsymbol{HO}.$$

We perform QR decomposition on $Y$ to obtain the orthogonal matrix $Q$ and the upper triangular matrix $R$:

$$\boldsymbol{Y}=\boldsymbol{QR}.$$

Next, we perform Singular Value Decomposition (SVD) on $Q$ to obtain the singular values $\Sigma$ and the singular value matrix $V$:

$$\boldsymbol{Q}=\boldsymbol{U}\boldsymbol{\Sigma}\boldsymbol{V}^{T}.$$

The low rank loss is approximated by the sum of the top $d$ (default 20) singular values

$$L_{norm}=\sum_{i=1}^{d} \sigma_{i}.$$

The overall loss function is then derived by taking the weighted sum of the similarity loss, spatial loss, and low rank loss.

$$Loss = L_{methods} + a * L_{Spatial} +b* L_{norm}.$$

Parameters $a$ and $b$ are both recommended to be set to 1 by default. We get the consensus similarity matrix $\boldsymbol{H}$ using the Adam optimizer to minimize the overall loss function $Loss$. $a$ and $b$ are both set to 1 by default.

**Spatial Domain Identification**

Finally, we perform clustering on the optimized consensus similarity matrix $\boldsymbol{H}$ using the spectral clustering algorithm. We construct the normalized Laplacian matrix $\boldsymbol{L}$ as follows:

$$\boldsymbol{L}=\boldsymbol{I}-\boldsymbol{D}^{-1/2}\boldsymbol{H}\boldsymbol{D}^{-1/2},$$

$$\boldsymbol{L}=\boldsymbol{U\Lambda}\boldsymbol{U}^{T},$$

where $\boldsymbol{I}$ is the identity matrix and $\boldsymbol{D}$ is the degree matrix. We perform eigenvalue decomposition on $\boldsymbol{L}$ and select the eigenvectors corresponding to the smallest $g$ eigenvalues to form the feature matrix $\boldsymbol{X}$. The default value for $g$ is 8. The matrix $\boldsymbol{X}$ is then used for K-means clustering to obtain the final spatial domain identification results.

# 2. Datasets

**Supplementary** **Table** **S1**. Summary of the datasets used in this study

| **Platform** | Tissue | Section | #Spots | #Genes |
| --- | --- | --- | --- | --- |
| **10x** **Visium** | Human breast cancer | N/A | 3798 | 36601 |
| **MERFISH** | Mouse hypothalamus | Bregma-0.04,  Bregma-0.09,  Bregma-0.14,  Bregma-0.19,  Bregma-0.24 | 5488,  5557,  5926,  5803,  5543 | 155 |
| **BaristaSeq** | Mouse primary visual area | Slice1,  Slice2,  Slice3 | 3390,  4491,  3545 | 79 |
| **STARmap** | Medial prefrontal cortex of the brain | BZ5,  BZ9,  BZ14 | 1049,  1053,  1088 | 166 |

We validate the performance of Space using SRT datasets from four different platforms (Supplementary Table S1), which can be downloaded from public repositories.

(1) [Human breast cancer](https://support.10xgenomics.com/spatial-gene-expression/datasets/1.1.0/V1_Breast_Cancer_Block_A_Section_1): This dataset contains 3,798 spots and 36,601 genes, along with 20 manually annotated regions.

(2) [Mouse hypothalamus](https://datadryad.org/stash/dataset/doi:10.5061/dryad.8t8s248): This dataset includes 5 tissue sections, with 8 regions manually annotated, and the number of spots varies from 5,488 to 5,926.

(3) [Mouse primary visual area](https://spacetx.github.io/data.html): This dataset comprises three slices, containing 3390, 4491, and 3545 spots, respectively, for a total of 79 genes. Manual annotation was performed on six visual cortex layers, ranging from VISP_I to VISP_VI, as well as the white matter region (VISpwm).

(4) [Mouse visual cortex](https://www.starmapresources.com/data): This dataset includes three tissue sections_BZ5, BZ9, and BZ14_which have spot counts of 1049, 1053, and 1088, respectively, amounting to a total of 166 genes. Manual annotation was conducted for four regions.

# 3. Functional Verification and Comparison Analysis

## 3.1 Human Breast Cancer Dataset


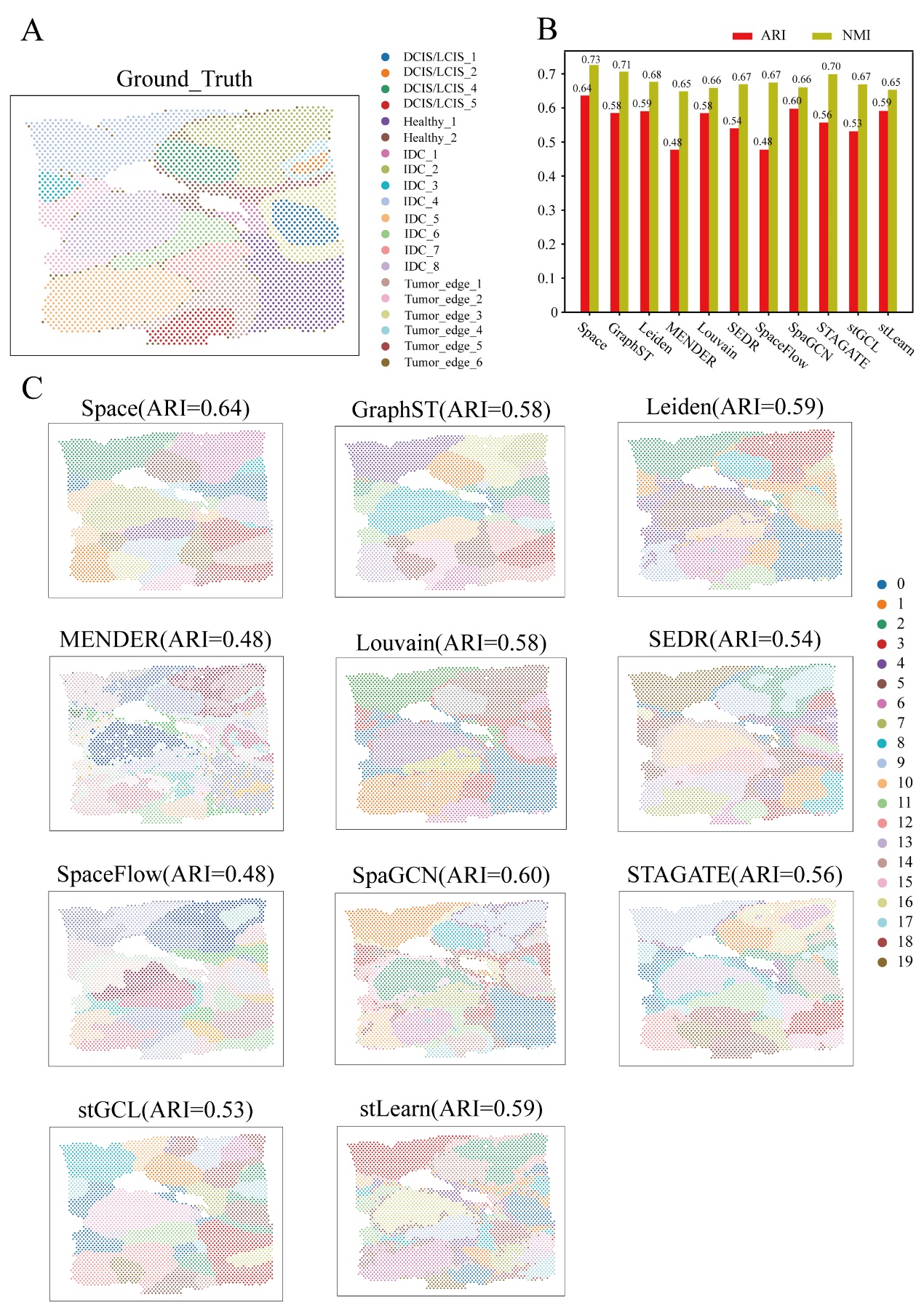


**Figure S1**. The results of human breast cancer dataset. (A) Manually annotated ground truth. (B) The bar plot of ARI and NMI value. (C) The clustering results of different methods.

We first evaluated the performance of Space using the human breast cancer dataset. In this dataset, Space achieved the highest ARI and NMI scores (Figure S1B). Space outperforms all other approaches in terms of boundary clarity and clustering effectiveness in the upper and upper-left regions of the image, including IDC_4, IDC_2, IDC_3, tumor_edge_2, and DCIS/LCIS_4. Moreover, the results are highly consistent with manual annotations. This indicates that Space can effectively integrate existing spatial domain recognition algorithms to produce superior clustering outcomes. For example, in the IDC_4 region located on the upper-left corner of the image, the clustering result from Space closely matches the Ground Truth, with clear boundaries. In contrast, MENDER, SpaceFlow, and stGCL exhibit significant discrepancies from the manual annotations in the overall delineation of the region. Their boundaries are unclear, and the separation between adjacent clusters is indistinct, failing to achieve effective clustering. Similarly, in the IDC_2 region in the upper-right corner of the image, methods such as graphST, SEDR, spaGCN, STAGATE, and stLearn divide this region into multiple categories. Additionally, Leiden and Louvain perform much worse than Space in delineating the cluster boundaries, leading to a mixture of points from different clusters at the edges.

## 3.2 Mouse Hypothalamus Dataset


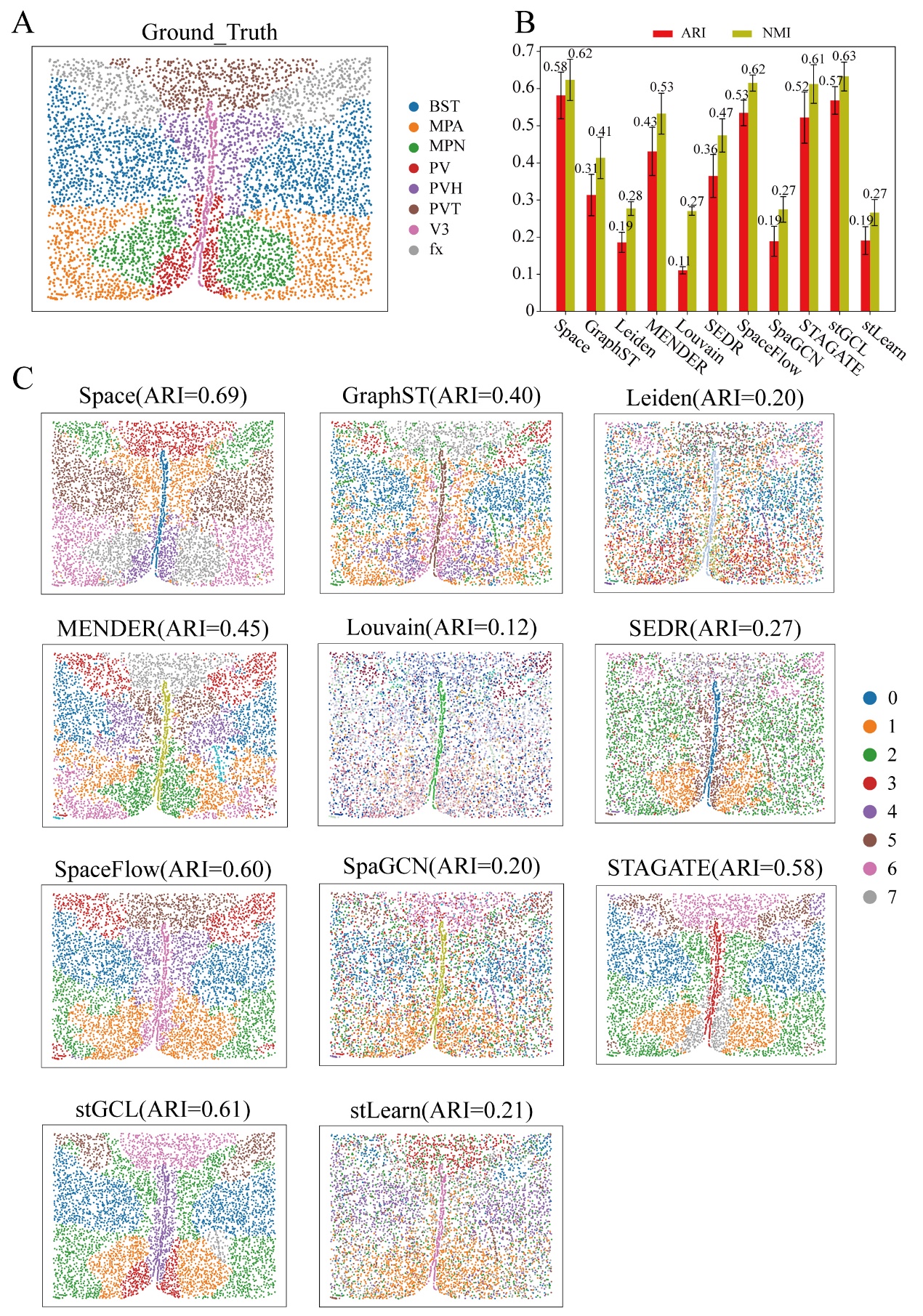


**Figure S2**. The results of mouse hypothalamus dataset. (A) Manually annotated ground truth. (B) The bar plot of ARI and NMI value. (C) The clustering results of different methods for slice Bregma-0.24.

Next, we conducted an analysis using mouse hypothalamus dataset generated by MERFISH. This dataset comprises five adjacent tissue sections from the anterior hypothalamic region of the mouse brain, denoted as Bregma-0.04 (5488 cells), Bregma-0.09 (5557 cells), Bregma-0.14 (5926 cells), Bregma-0.19 (5803 cells), and Bregma-0.24 (5543 cells). Cells in all tissue sections were categorized into eight distinct structures: V3, BST, fx, MPA, MPN, PV, PVH, and PVT.

We performed a detailed analysis of the Bregma-0.24 section and conduct a multi-slice analysis across all five sections. Using Space, regions such as BST, MPA, and MPN show excellent continuity and smoothness in their segmentation. In contrast, stLearn, Leiden, spaGCN, Louvain, and graphST exhibit significant overlap and blurred boundaries between clustering domains. SEDR incorrectly groups most regions into a single category. Although STAGATE, SpaceFlow, and stGCL demonstrate better visual outcomes in some areas, they still encounter problems in clustering distinct areas. For instance, the clustering results of STAGATE in the fornix region are confused with other categories, and both SpaceFlow and stGCL make classification errors in the medial preoptic area. Ultimately, when considering all five MERFISH sections, the Space achieves an average ARI of 0.58 and an average NMI of 0.62, clearly outperforming the other methods.

## 3.3 Mouse Primary Visual Area Dataset


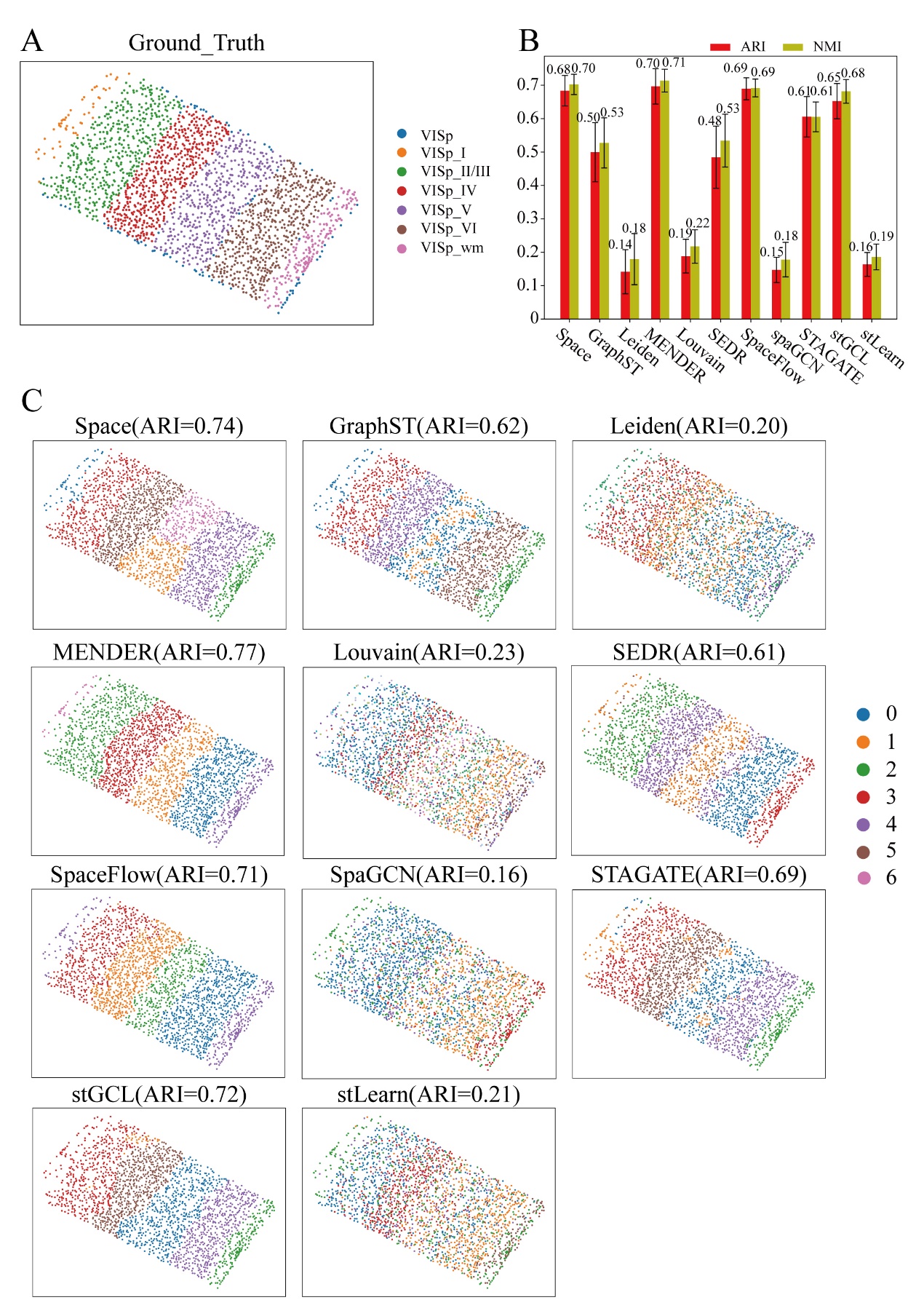


**Figure S3**. The results of mouse primary visual area dataset. (A) Manually annotated ground truth. (B) The bar plot of ARI and NMI value. (C) The clustering results of different methods for slice 2.

We further evaluated the performance of Space using the BaristaSeq dataset. This dataset, measured using BaristaSeq technology, consists of three slices from the primary visual area of a mouse, comprising a total of 11,426 cells and 79 genes. We used slice 2 from the BaristaSeq dataset to illustrate the spatial domain identification performance.

In this dataset, Leiden, Louvain, and stLearn exhibit significant cluster mixing, with unclear boundaries between layers. GraphST and STAGATE show issues with spot confusion and blurred clustering boundaries in the VISp_V region. Although SpaceFlow and stGCL are able to identify the main tissue regions, the boundaries between these regions are not accurately defined. Specifically, SpaceFlow fails to clearly distinguish between the VISp_V and VISp_VI layers. Similarly, stGCL and MENDER encounter boundary inaccuracies when classifying the VISp_IV and VISp_II/III layers. In the VISp_wm layer, several baseline methods demonstrate significant cluster mixing, with unclear boundaries between the WM domain and the VISp_VI layer. Among all methods, Space achieves the highest ARI score on a single slice, reaching 0.74. When considering all three slices, Space demonstrates relatively stable and reliable performance, with an average ARI score of 0.69 and an average NMI score of 0.71.

## 3.3 Medial Prefrontal Cortex of the Brain


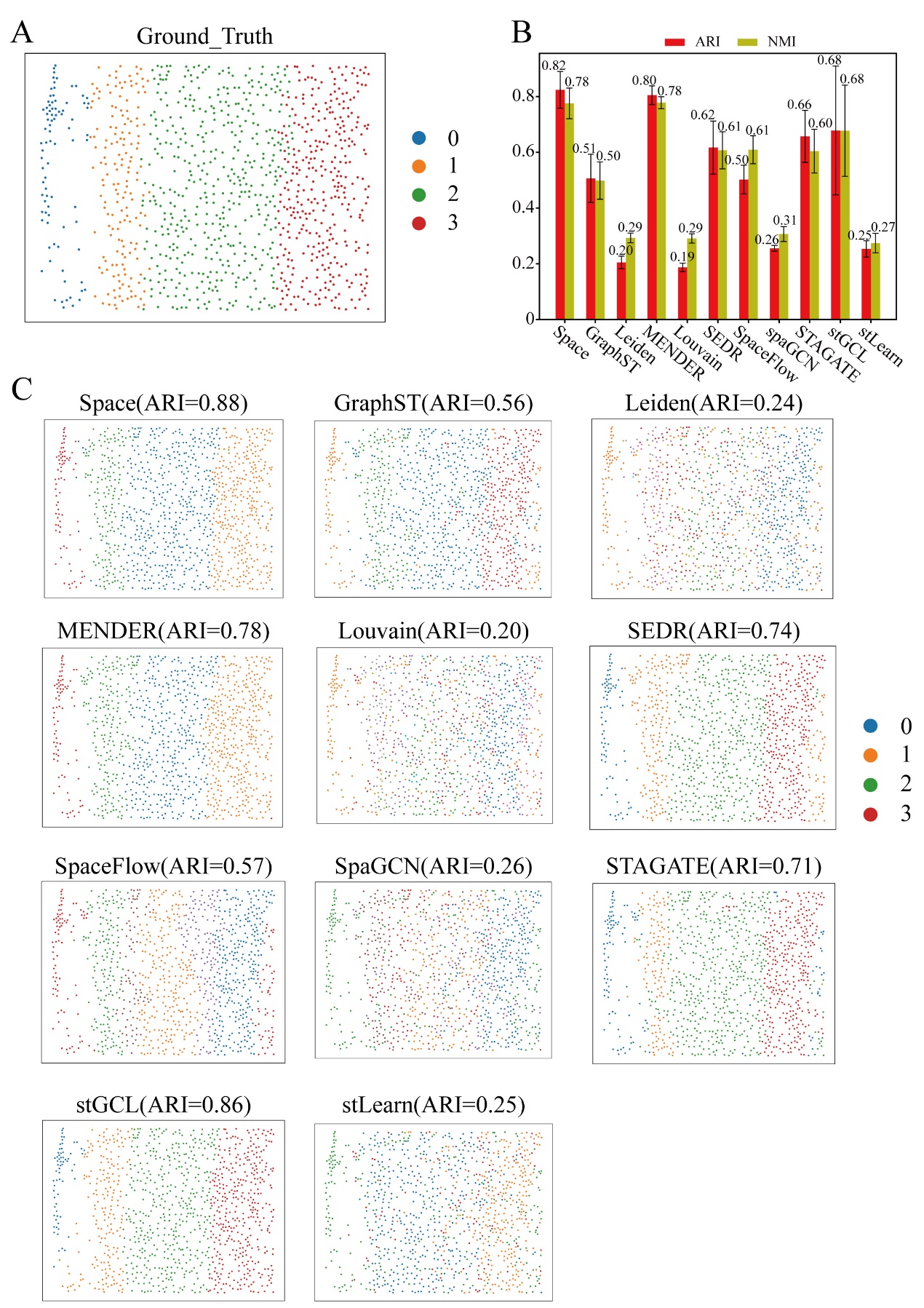


**Figure S4**. The results of medial prefrontal cortex of the brain. (A) Manually annotated ground truth. (B) The bar plot of ARI and NMI value. (C) The clustering results of different methods for slice BZ5.

Then, we conducted an analysis using the dataset generated by STARmap. This dataset consists of three tissue slices from the medial prefrontal cortex (mPFC) of the mouse brain. The mPFC is a medial region of the prefrontal cortex and plays a crucial role in cognitive functions, emotional regulation, decision-making, and behavioral control. The three tissue slices are BZ5 (1049 cells), BZ9 (1053 cells), and BZ14 (1088 cells), each with expression measurements for 166 common genes.

We performed both single-slice analysis on BZ5 and multi-slice analysis across all three slices. The results show that the domains identified by Space closely match the ground truth. Space correctly identifies four distinct layers and produces smoother segmentation. In contrast, methods such as graphST, Leiden, Louvain, spaGCN, and stLearn produce unclear domain boundaries with spot confusion. SpaceFlow incorrectly splits the second layer into multiple layers. SEDR and STAGATE exhibit boundary blurring issues in the clustering of the third layer, leading to suboptimal clustering performance. While MENDER and stGCL can identify the main regions, the boundaries between domains are not precise enough. For example, MENDER performs slightly worse than Space in clustering at the boundary between the second and third layers, resulting in less clear boundaries. Similarly, stGCL shows clustering confusion at the boundary between layer 0 and layer 1, mistakenly clustering part of layer 0 into layer 1. The ARI score of the Space reaches as high as 0.88 on a single slice. When considering all three STARmap slices, the average ARI score of Space is 0.82, significantly higher than almost all other methods. In summary, Space demonstrates outstanding and reliable performance in clustering tasks on the STARmap dataset.

# 4. Computational Cost Analysis

To evaluate the feasibility of Space in real-world applications, we conducted a detailed assessment of its runtime and computational resource requirements. Specifically, we measured the execution time and GPU memory consumption of Space on simulated datasets. As illustrated in Figure S5, both execution time and GPU memory usage scale linearly with the number of spots. Notably, Space efficiently processed a dataset containing 20,000 spots in just 13 minutes, with GPU memory usage of approximately 24 GB (tested on an Intel Xeon Gold 6226R CPU, 128 GB RAM, and an NVIDIA RTX A6000 GPU). These results clearly demonstrate that Space can effectively handle large-scale datasets, providing users with valuable insights to assess its practical applicability.


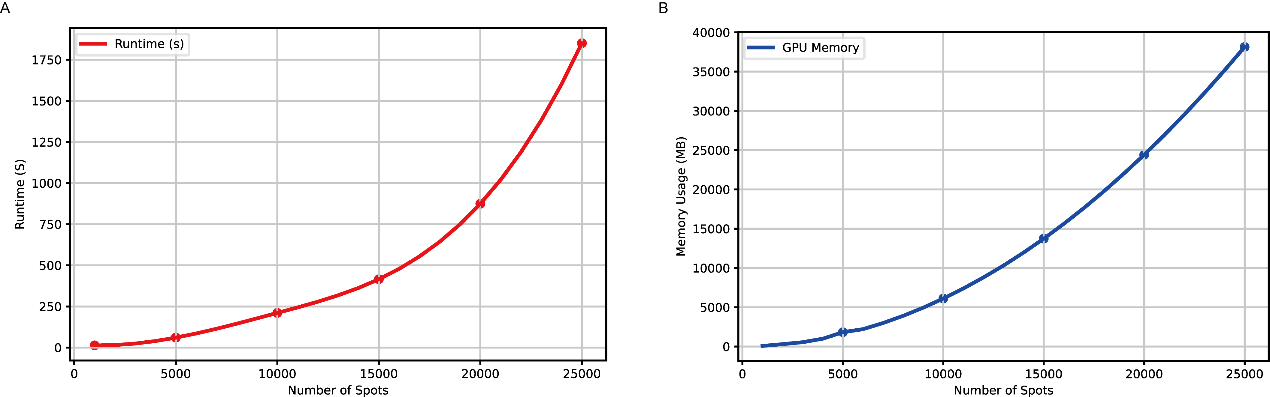


**Figure S5**. The computational cost of Space. (A) The running time of Space on the simulated dataset. (B) GPU memory usage of Space on simulated datasets.

# 5. Reference

Blondel,V.D. *et al.* (2008) Fast unfolding of communities in large networks. *J. Stat. Mech.*, **2008**, P10008.

Dong,K. and Zhang,S. (2022) Deciphering spatial domains from spatially resolved transcriptomics with an adaptive graph attention auto-encoder. *Nat Commun*, **13**, 1739.

Hu,J. *et al.* (2021) SpaGCN: Integrating gene expression, spatial location and histology to identify spatial domains and spatially variable genes by graph convolutional network. *Nat Methods*, **18**, 1342–1351.

Long,Y. *et al.* (2023) Spatially informed clustering, integration, and deconvolution of spatial transcriptomics with GraphST. *Nat Commun*, **14**, 1155.

Pham,D. *et al.* (2020) stLearn: integrating spatial location, tissue morphology and gene expression to find cell types, cell-cell interactions and spatial trajectories within undissociated tissues. *bioRxiv*, 2020.05.31.125658.

Ren,H. *et al.* (2022) Identifying multicellular spatiotemporal organization of cells with SpaceFlow. *Nat Commun*, **13**, 4076.

Traag,V.A. *et al.* (2019) From Louvain to Leiden: guaranteeing well-connected communities. *Sci Rep*, **9**, 5233.

Xu,H. *et al.* (2024) Unsupervised spatially embedded deep representation of spatial transcriptomics. *Genome Medicine*, **16**, 12.

Yu,N. *et al.* (2023) stGCL: A versatile cross-modality fusion method based on multi-modal graph contrastive learning for spatial transcriptomics. *bioRxiv*, 2023.12.10.571025.

Yuan,Z. (2024) MENDER: fast and scalable tissue structure identification in spatial omics data. *Nat Commun*, **15**, 207.
